# Aging Time Prediction Model Analysis and Numerical Simulation of Random Degradation Equipment Based on Big Data Linkage Technology

**DOI:** 10.1155/2022/4278849

**Published:** 2022-08-23

**Authors:** Chen Ye, Xuefeng Peng

**Affiliations:** College of Science and Technology Ningbo University, Cixi 315300, China

## Abstract

In this study, we focus on the relevance of remaining life prediction of randomly degraded equipment in the context of big data monitoring and the core issue of quantifying uncertainty in remaining life prediction. We analyze the limitations and common problems of current research. To address the limitations and common problems, a solution for predicting the remaining life of randomly degraded devices with multisource sensing monitoring in the context of big data is proposed, and the feasibility and effectiveness of the idea are verified using battery data. Finally, multiple machine learning methods, such as support vector machines, random forests, recurrent neural networks, and convolutional neural networks, are combined to predict the remaining life of batteries, and these four machine learning methods perform well in the work of battery remaining life prediction and solve the key scientific problems.

## 1. Introduction

**Figure 1:**
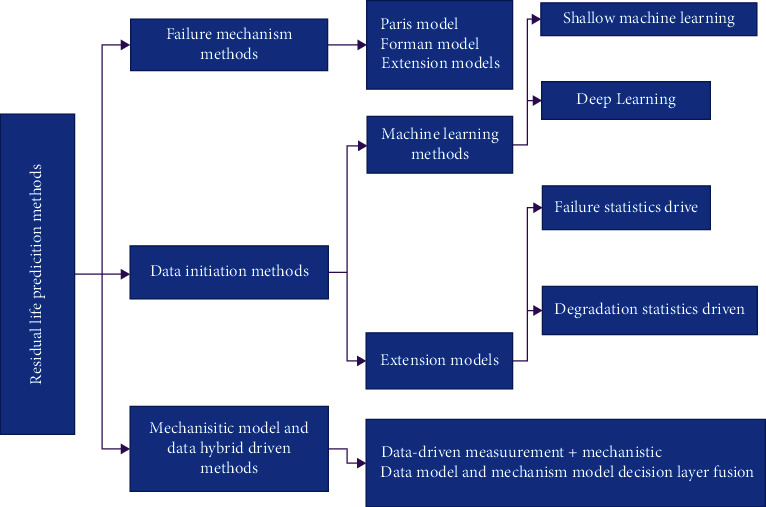
The methodology of remaining useful life prediction.

The safe and reliable operation of equipment is highly dependent on healthy performance; however, equipment will gradually age during operation, which in turn will lead to reduced performance or even failure, further causing system downtime or even catastrophic accidents. Modern equipment such as high-speed trains [1], missile weapons [2, 3], industrial robots [4, 5], petrochemical equipment, and other modern equipment are gradually becoming larger, more diversified, and integrated at the same time as their functions continue to improve. The service process is affected by variable environment, variable load, variable working conditions, large disturbances, and strong impact; the overall and key components performance will inevitably degrade; once the equipment performance degradation is caused by the final failure, it will cause huge casualties and property damage. The investigation of the July 2017 crash of a US Marine Corps KC-130 transport aircraft in Mississippi, USA, in which all 16 servicemen on board were killed, showed that the degradation of the engine propeller was the main cause of the crash. Therefore, it is very necessary to take measures to ensure the safety and reliability of the equipment. Engineering practice shows that predictive and health management (PHM) techniques can reduce maintenance costs, improve equipment reliability and safety, and reduce the risk of failure events. Among them, the remaining life prediction is the key to connect the system operation state information sensing and personalized and accurate health management based on the operation state, which has been developed significantly in the past ten years or so. The failure mechanism analysis-based methods mainly construct mathematical models describing the failure mechanism of equipment and combine the empirical knowledge of specific equipment and defect growth equation to achieve the remaining life prediction of equipment. Due to the complexity of the actual engineering equipment and the diversity of tasks and operating environments, the evolution of its health state is often difficult to model physically and mechanically or the cost of obtaining a failure mechanism model is too high. Therefore, the data-driven remaining life prediction technology has become the frontier of international research in the field of reliability engineering and automation technology and has been greatly developed in the past decade or so. The data-driven remaining life prediction techniques have become the frontier of international research in the field of reliability engineering and automation technology. In summary, effective remaining life prediction is of great importance to improve the reliability and safety of equipment in use. Various methods of remaining service life prediction are shown in Figure 1.

Along with the fast development of sophisticated sensing science, the health status of engineering equipment [6, 7] is becoming more and more abundant, which provides more possibilities to obtain big data for equipment operation monitoring. The problem of big data processing for predicting the remaining life of randomly degraded devices has received widespread attention, and related technologies are flourishing. The main objective of this study is to analyze the current state of development of residual life prediction technology, explore the key issues and new directions in this field, and predict the residual life of randomly degraded equipment by four machine learning algorithms for predicting data in engineering practice.

## 2. Overview of the Remaining Life Prediction Problem with Big Data

In the past decade, with the swift increase in the rise and popularity of wireless sensing, the Internet of Things [8, 9], and other technologies, all kinds of sensors are like a giant neural network closely spaced inside the device, sensing every move of the device in real-time and driving the remaining life prediction into the “big data” era. The remaining life prediction enters the era of “big data.” For example, military equipment plays an irreplaceable role in the safe and reliable operation of its subsystems. The safe and reliable operation of its subsystems is crucial and must be protected by theories and methods of condition monitoring, remaining life prediction [10], and predictive maintenance. Modern aviation engines can generate hundreds of sensor information every 10 milliseconds, and each flight can generate terabytes of operational monitoring data; modern industrial manufacturing lines have tens of thousands of sensors to monitor the operational process and product quality information of industrial equipment, such as large industrial robot manufacturers use cloud platforms to monitor millions of industrial robots and obtain the speed, angle, position, temperature, and vibration of each robot joint real-time signals.

With the rapid development of information technology and sensing technology, data-driven remaining life prediction techniques have been widely and efficiently used due to their wide range of applications. Machine learning methods and data statistic-driven methods have been widely researched and developed in the field of remaining life prediction by academia and industry. These techniques have been used in missile weapons, aerospace, and wind power generation. Although data-driven prediction of remaining life of stochastic degraded equipment, typified by machine learning methods and statistical data-driven methods, has been heavily researched and flourished, there is still no systematic and effective solution to the core problem of predicting remaining life of stochastic degraded equipment in the context of big data and quantifying its prediction uncertainty. The main problems are the inability of statistical data-driven approaches to handle big data and the inability of machine learning approaches to quantify the prediction uncertainty. Therefore, it is important to study the remaining life prediction under machine learning methods and analyze the bottlenecks of current research to promote the development of remaining life prediction techniques for randomly degraded devices.

## 3. Hybrid Mechanistic Model and Data-Driven Remaining Life Prediction

The mechanistic model-based approach focuses on the construction of a parametric mathematical model based on the failure mechanism parametric mathematical model describing the degradation process of the equipment, combined with the equipment's design test data or empirical knowledge to identify the parameters of the mathematical model, and the model parameters are then updated based on the condition monitoring data to achieve the remaining life of the equipment. The remaining life of the equipment is predicted based on the condition monitoring data. Typical parameter identification and updating methods include Kalman filtering [11, 12], particle filtering [13, 14], and Bayesian methods [15–17]. The common mechanistic models used for the remaining life prediction include the Paris model, Forman model, and various improvements and extensions based on them, mainly to describe the crack expansion and laminar crack growth [18]. For example, Li et al. developed a mapping between defect growth rate and defect area and material constants to predict the remaining life of rolling. Liang et al. studied the adaptive prediction method of remaining service life of ball bearings based on the Paris model, even in the absence of a priori information and the growth of defects. Even in the absence of a priori information and time-varying defect growth, reliable prediction results were obtained. Choi et al. proposed a life model for lamellar crack growth in rolling contact by considering the phenomenon of lamellar crack growth due to crack formation and abrasive wear. The methods mentioned above are not combined with the real-time monitoring of actual operating equipment, so it is difficult to accurately reflect the current operating status of the equipment, especially when the operating environment and operating conditions change. When the running conditions and environmental change, if the model cannot be updated with the real-time monitoring data, large prediction deviations will occur.

In order to make the mechanistic models better model the performance evolution of individual equipment in actual service, mixing the equipment's real-time operation monitoring data with the mechanistic models will help to achieve the improvement of the remaining life prediction accuracy. Therefore, the mixture of mechanistic models and data-driven remaining life prediction methods has received more attention and development, which is described in the review paper. Recent studies in this area include those by Liao and Köttig et al. and Wang et al. where a hybrid mechanistic model and data-driven remaining life prediction method for lithium battery systems and rotating mechanical devices are proposed, respectively. These hybrid-driven methods for residual life forecasting can be divided into two groups, depending on the different ways of implementing the hybrid mechanic's models and data.

1. The measurement model of the degradation state depicted by the mechanics model is built based on the monitoring data, the parameters of the degradation state and the mechanics model are estimated using the Kalman filter and particle filter, and finally, the remaining life of the equipment is predicted by the mechanics model.
2. First, the measurement model of the degradation state based on the data and the mechanics model are performed separately, and then, the remaining life of the equipment is predicted by the mechanics model. First, the remaining life of the equipment is predicted based on the data and mechanics models, and then, the decision layer fusion method is used to achieve the integrated prediction of the remaining life based on the data and mechanics models.

The first method can take into account the fact that the degradation state is difficult to measure directly and can achieve the estimation of the implied degradation state and update the parameters of the mechanics model while considering the measurement noise of the monitoring data so that the final prediction results can reflect the current actual state of the equipment more accurately. The second method is relatively simple and independent, and the decision-making layer can be incorporated into various forms, which can integrate the advantages of multiple methods and help improve the robustness of the prediction results.

## 4. Remaining Life Prediction Based on Machine Learning

The main idea of machine learning-based equipment remaining life prediction is by fitting the evolution of performance variables by machine learning and rolling out to the failure threshold to predict the time to failure or by directly establishing the mapping between monitoring data and time to failure to achieve end-to-end prediction. End-to-end prediction by mapping monitoring data to failure time is based on the predicted failure time minus the current operation time to obtain the remaining predicted lifetime. Pei Hong et al. and Khan et al. reviewed the effectiveness of machine learning methods and deep learning methods in predicting the failure time of machine learning methods and deep learning methods in the field of remaining life prediction and health management. To distinguish from the works presented in the above reviews, this work focuses on the latest research progress and the realistic needs of residual life prediction with big data. In this study, we focus on the development and problems of machine learning-based remaining life prediction technology in the context of the latest research progress and the realistic needs of remaining life prediction under large data.

Typical methods and specific developments of remaining life prediction by shallow machine learning-based neural networks and support vector machines are described in the following sections.

### 4.1. Neural Networks

The neural network is a learning network that simulates the organization and information processing mechanism of the human central nervous system, mainly consisting of input, hidden, and output layers. Neural networks have the advantages of self-learning, self-organization, self-adaptation, and strong nonlinear mapping fitting ability and therefore have received a lot of attention in the field of equipment remaining life prediction. As early as 2004, Gebraeel et al. used a single hidden layer feedforward neural network for vibration signal modeling of mechanical equipment to achieve remaining life prediction by extrapolation to a failure threshold. Mahamad et al. used the improved feedforward neural network training algorithm for life prediction of rotating machinery. Lim et al. extracted degradation indicators with local trends from multisource monitoring data by using the characteristic time-series histogram method and then fed these degradation indicators into a multilayer perceptron to predict the remaining life of an aeroengine. Drouillet et al. used a single hidden layer feedforward neural network for residual life prediction of high-speed milling cutters. Ahmadzade used a multilayer perceptron to predict the residual life of a grinder. Zhang et al. constructed a blower remaining life prediction model based on the wavelet packet decomposition, fast Fourier transform, and back propagation neural network. Xu Donghui et al. proposed a multiclass neural network combination prediction method using two single prediction models, the improved Elman neural network and the nonlinear autoregressive neural network, and a nonlinear combination of the predicted values of the two single models with the help of the radial basis function neural network to achieve the remaining life prediction. Yang et al. investigated a lithium battery life prediction method based on a combined autoregressive moving average and backward propagation neural network model, which effectively combined the advantages of both short-term prediction and nonlinear fitting. In a recent study, Bektas et al. proposed a neural network and similarity-based method for remaining lifetime prediction by introducing data preprocessing techniques, such as sensor selection, data normalization, and feature extraction, and using the preprocessed data for training neural networks. Li and Terpenny et al. used monitoring data to train several neural networks and proposed an integrated network-based method for remaining life prediction based on the idea of weighted average.

### 4.2. Support Vector Machines

Support vector machines were first proposed by Cortes and Vapnik in 1995 and have received much attention in the field of machine learning for small samples and high-dimensional data. The main principle is to first map multidimensional input vectors to a high-dimensional feature space by a nonlinear transformation and then construct the optimal hyperplane in the high-dimensional feature space to achieve sample classification or regression. Support vector machines have been widely used for remaining life prediction of equipment because of their ability to avoid the “dimensional catastrophe” problem and their good generalization ability.

The SVM is looking to discover the furthest point to the hypoplane for each class of sample points, that is, to discover the hypoplane of the largest interval. The arbitrary hyperplane can be characterized by the following linear equation.

(1) $$\begin{array}{c} w^{T}x+b=0. \end{array}$$

The formula for the distance from the point (*x*,*y*) in two dimensions to the line *Ax* + *By* + *c* = 0 is

(2) $$\begin{array}{c} \frac{Ax+By+c}{\sqrt{A^{2}+B^{2}}}. \end{array}$$

After expanding to the n-dimensional space, the distance from the point *x*=(*x*_1_, *x*_2_, ..., *x*_*n*_) to the line *w*^*T*^*x*+*b*=0 is

(3) $$\begin{array}{c} \frac{\left|w^{T}x+b\right|}{\left\|w\right\|},\\ \left\|m\right\|=\sqrt{w_{1}^{2}+...+w_{n}^{2}}. \end{array}$$

**Figure 2:**
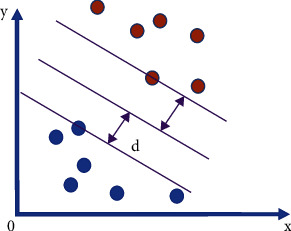
Schematic diagram of the hyperplane.

As shown in the figure, by the definition of support vector, we are aware that the range of the support vector to the hyperplane is *d* and the range of distance from other points to the hyperplane is greater than *d*.  Figure 2 shows the schematic diagram of the hyperplane.

By optimization, we can obtain the following final equation:

(4) $$\begin{array}{c} \min\frac{1}{2}{\left\|m\right\|}^{2}s\text{.t}.y_{\partial }\left(w^{T}x_{i}+b\right)>1. \end{array}$$

### 4.3. Random Forest

In an intelligent algorithm, a random forest (RF) is a categorizer including multiple decision trees whose outcome classes are dictated from multiple classes of the output of the respective trees. To explain it intuitively, each decision tree is a classifier (suppose we are now solving a classification problem), and therefore, for an input example, *N* trees will yield *N* classification results. The random forest integrates all the categorization polls and takes the category with the most votes as the outcome, which represents one of the simplest bagging thoughts to date.

### 4.4. Recurrent Neural Network

The RNN is a particular neural network architecture that is built on the fact that a human's perception is grounded in past and memory experiences. It takes into account not only the input from the previous moment but also gives the network a “memory” function for prior content.

**Figure 3:**
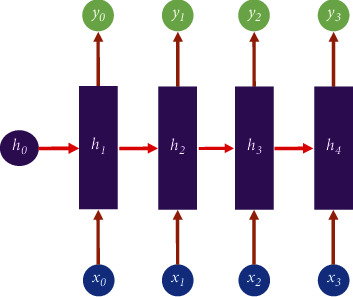
The structure of the RNN.

The RNN is known as a recurrent neural network because the present output of a series is also related to the prior output. It is represented as a network that remembers the previous information and applies it to the computation of the current output, i.e., the hidden layers are no longer disconnected from each other, but connected, and the nodes of the hidden layers include not only the output of the input layer but also the output of the hidden layer at the former moment.  Figure 3 illustrates the common structure of RNNs. Recurrent neural networks have applications in natural language processing (NLP), such as speech recognition, language modeling, and machine translation, and are also used in various types of time series forecasting.

In Figure 3, *h* is the hidden state, *x* is the input, *y* is the output, and *h*_*0*_ is the initial hidden state and are calculated as follows.

(5) $$\begin{array}{c} h_{t}=f\left(Ux_{t}+W\left(h_{t-1}+b\right)\right),\\ y_{t}=\text{Softmax}\left(Vh_{t-1}+c\right). \end{array}$$

### 4.5. Convolutional Neural Networks

**Figure 4:**
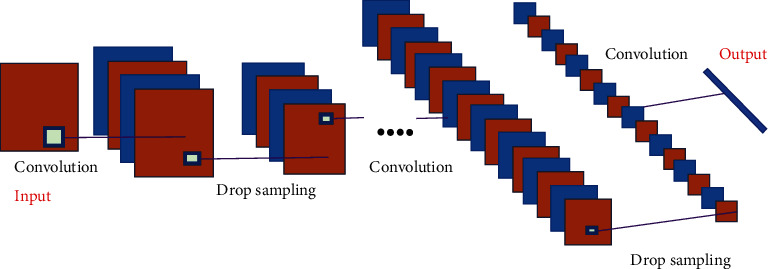
Schematic diagram of the structure of a convolutional neural network.

Convolutional neural networks (CNNs) usually consist of the following layers. Each layer of a convolutional neural network is made up of multiple convolutional units, and the parameters of each convolutional unit are optimized by a back propagation method. The aim of the convolutional operation is to select various features from the input. The initial convolutional layers may extract only some low-level features, such as fringes, lines, and curves, while further layers of the network can repeatedly extract more sophisticated features from the low-level characteristics. Linear rectification layer uses a linear rectification of the neural activation function. The pooling layer, which usually follows the convolutional layer to obtain features of large dimensionality, slices the characteristics into regions and takes their maximum or average value to obtain novel, lower dimensional features.  Figure 4 shows a schematic diagram of the structure of the convolutional neural network.

The convolution process is the sum of all the weights of the kernel and their corresponding element luminance on the input image, which can be expressed as

(6) $$\begin{array}{c} {\text{conv}}_{x,y}=\sum_{i}^{p*q}w_{i}v_{i}. \end{array}$$

After the activation function, the result is obtained as

(7) $$\begin{array}{c} z_{x,y}=h\left(\sum_{p*q}^{I}w_{i}v_{i}+b\right). \end{array}$$

The commonly used activation functions are as follows.

Linear rectification unit (ReLU):

(8) $$\begin{array}{c} h\left(z\right)=\max\left(0,z\right). \end{array}$$

Sigmoid function:

(9) $$\begin{array}{c} h\left(z\right)=\frac{1}{\left(1+e^{-z}\right)}. \end{array}$$

Tanh function:

(10) $$\begin{array}{c} h\left(z\right)=\tanh\left(z\right). \end{array}$$

## 5. Experimental Verification

Using the monitoring data collected during the charging and discharging cycle of the battery, the operating status information of the battery is extracted to further predict the remaining service life of the battery equipment, and the life assessment and intelligent management are carried out through the life supervision platform of electromechanical equipment. Battery data selection: the publicly available lithium battery dataset from the NASA PCoE Research Center is used for model validation, and this battery dataset is used to perform cyclic charge/discharge experiments on lithium batteries. The collected experimental environment and conditions of the battery device during the charging/discharging process are shown in Table 1.  Table 2 shows the specific monitoring parameters.

After the specific parameters of the prediction model are determined, the life feature vectors extracted in the previous section are used as the input data of the forecasting model to train the residual life prediction model of the battery device. First, the extracted lifetime feature vectors are normalized, and the data collection is split into a training set and a test set in the ratio of 3 : 1. The training set is applied to train the prediction network, and the test set is applied to verify the training effect of the prediction model.

**Figure 5:**
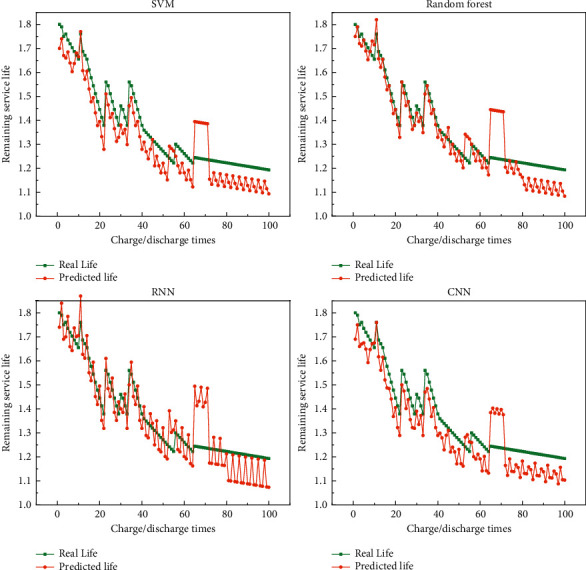
Remaining life prediction results of B5 batteries: (a) SVM-based prediction results; (b) random forest-based prediction results; (c) RNN-based prediction results; (d) CNN-based prediction results.

**Figure 6:**
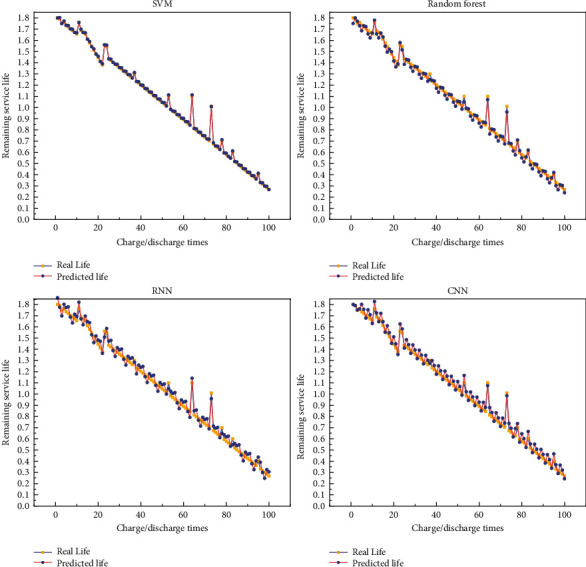
Remaining life prediction results of B6 batteries: (a) SVM-based prediction results; (b) random forest-based prediction results; (c) RNN-based prediction results; (d) CNN-based prediction results.

**Figure 7:**
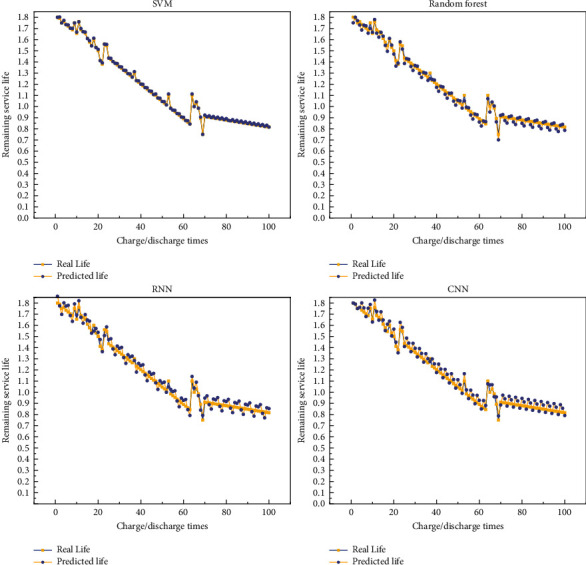
Remaining life prediction results of B7 batteries: (a) SVM-based prediction results; (b) random forest-based prediction results; (c) RNN-based prediction results; (d) CNN-based prediction results.

In the battery dataset, the battery is cycled through charge/discharge experiments until the battery capacity drops to a fixed value. As the battery performance decreases, each cycle has a different charge/discharge time, and therefore, the nodes for collecting monitoring parameters within each cycle are different. The collected raw monitoring data are first preprocessed and the data are integrated with each cycle as the minimum period to obtain the input dataset for the life prediction network. The monitoring data of four groups of lithium batteries, B5, B6, B7, and B18, are used as experimental samples, and the cycle numbers of the four battery devices are 168, 168, 168, and 132, respectively. Then, the number of datasets of the battery devices corresponds to the cycle numbers. In the ratio of 3 : 1, the monitoring data of B6, B7, and B18 battery devices are used to create a training set for training the remaining life prediction method proposed in this chapter, and the processed monitoring parameters of B5 battery devices are used to create a test set for testing the final prediction model effect, focusing on showing the remaining life prediction results of B5, B6, and B7 battery.  Figure 5 shows the remaining life prediction results of B5 batteries.  Figure 6 shows the remaining life prediction results of B6 batteries.  Figure 7 shows the remaining life prediction results of B7 batteries.

We can see from the figure that for the remaining life prediction results of the B5 battery, the prediction results are not satisfied no matter which model is used. However, among the four models, the prediction results based on random forest are relatively better, and for the B5 battery, the prediction results of random forest are more instructive. The prediction results of the other three models can be significantly biased. For the remaining life prediction results of the B6 battery, the SVM-based prediction results are the best, and it can reflect the results well, but random forest, RNN, and CNN can also have good prediction results. For the remaining life prediction result of the B7 battery, the result is the same as B6, and all four prediction models have good results. In summary, it can be seen that all four models perform well in predicting the battery. Although we did not describe the prediction of battery life quantitatively, we can observe the effect of these prediction models from these pictures. In future engineering applications, our recommendation is to use deep neural networks for predicting battery life. Because we do not have a lot of data, if we increase the experimental data, the effect of traditional machine learning methods must be inferior to deep learning methods.

## 6. Results and Discussion

In this study, we focus on the realistic need for residual life prediction of randomly degraded devices in the context of big data monitoring and the central issue of quantifying uncertainty in residual life prediction. In response to the current limitations and common problems, a solution based on numerical-model linkage for the remaining life of randomly degraded devices in the context of big data is proposed, and the feasibility and effectiveness of the idea are verified using battery monitoring data. Finally, machine learning methods such as knotted support vector machines, random forests, recurrent neural networks, and convolutional neural networks are used to predict the remaining battery life, and these four machine learning methods perform well in the battery remaining life prediction work and solve the key scientific problems. However, we do not have a large amount of data in our experiments, and in future work, we will collect real-time data from multiple random degradation devices to verify the effectiveness of the algorithms. After increasing the amount of data again, we believe that the prediction effect of traditional machine learning-based methods will be affected, and the effect of deep neural network-based models, such as the RNN and CNN, will remain good. This is because deep network models will have better adaptability and robustness for processing large data.

## Figures and Tables

**Table 1:** Experimental environment and conditions.

Environment and conditions	Content description
Environmental testing room	Temperature 24°C, charging, discharging, and impedance are the 3 kinds of working conditions
Charging process	Charge at a constant current of 1.5 A as long as the battery voltage reaches 4.2 V
Discharge process	Discharge with a constant current of 2 A until the battery voltage drops to the minimum value
Experimental termination conditions	When the rated capacitance of the battery (from 2.4 Ahr to 1.4 Ahr) drops to 30%

**Table 2:** Specific monitoring parameters.

No.	Unit
Voltage_measured	V
Current_measured	A
Temperature_measured	°C
Current_charge	A
Voltage_charge	V
Current_load	A
Voltage_load	V
Time	s

## Data Availability

The data used to support the findings of this study are available from the corresponding author upon request.

## Abbreviations

NN: Neural networks
SVM: Support vector machine
RF: Random forest
RNN: Recurrent neural network
CNN: Convolutional neural network.
